# Differences in all-cause and cause-specific mortality due to external causes and suicide between young adult refugees, non-refugee immigrants and Swedish-born young adults: The role of education and migration-related factors

**DOI:** 10.1371/journal.pone.0279096

**Published:** 2022-12-20

**Authors:** Marlene Stratmann, Emma Björkenstam, Thomas E. Dorner, Lingjing Chen, Magnus Helgesson, Alexis E. Cullen, Ellenor Mittendorfer-Rutz

**Affiliations:** 1 Division of Insurance Medicine, Department of Clinical Neuroscience, Karolinska Institutet, Stockholm, Sweden; 2 Faculty of Health, Department of Health Sciences, Karlstad University, Science and Technology, Karlstad, Sweden; 3 Department of Neuroscience, Uppsala University, Uppsala, Sweden; 4 Department of Community Health Sciences, Fielding School of Public Health and California Center for Population Research, University of California Los Angeles, Los Angeles, CA, United States of America; 5 Department of Social and Preventive Medicine, Medical University of Vienna, Centre for Public Health, Vienna, Austria; 6 Department of Psychosis Studies, King’s College London, Institute of Psychiatry, Psychology & Neuroscience, London, United Kingdom; Aarhus University: Aarhus Universitet, DENMARK

## Abstract

**Background:**

International migration has increased during the past years and little is known about the mortality of young adult immigrants and refugees that came to Sweden as children. This study aimed to investigate 1) the risk of all-cause and cause-specific mortality in young accompanied and unaccompanied refugees and non-refugee immigrants compared to Swedish born individuals; and 2) to determine the role of educational level and migrations-related factors in these associations.

**Methods:**

This register linkage study is based on 682,358 individuals (633,167 Swedish-born, 2,163 unaccompanied and 25,658 accompanied refugees and 21,370 non-refugee immigrants) 19–25 years old, who resided in Sweden 31.12.2004. Outcomes were all-cause mortality and mortality due to suicide and external causes. Hazard ratios (HR) and 95% confidence intervals (CI) were calculated using Cox regression models with a maximum follow-up to 2016.

**Results:**

After adjusting for covariates, all-cause mortality was significantly lower in non-refugee immigrants (aHR 0.70, 95% CI 0.59–0.84) and refugees (aHR 0.76, 95% CI 0.65–0.88) compared to Swedish-born individuals. The same direction of association was observed for mortality due to suicide and external causes. No differences between accompanied and unaccompanied refugees were found. Risk estimates for all migrant groups varied with educational level, duration of residency, age at arrival and country of birth. Further, the mortality risk of migrants arriving in Sweden before the age of 6 years did not significantly differ from the risk of their Swedish-born peers. Low education was a considerable risk factor.

**Conclusion:**

In general, young adult refugees and non-refugee immigrants have a lower risk of all-cause and cause-specific mortality than Swedish-born individuals. The identified migrant groups with higher mortality risk need specific attention.

## Introduction

International migration has increased during the last decade and has already surpassed migration estimates for the year 2050 [1]. According to a report from the World Health Organisation, 272 million of the world’s population are migrants, with 14% younger than 20 years of age [1]. In 2015, Sweden received the largest number of refugees seeking asylum per capita (16.7 per 1000) in Europe [2]. One in three of these refugees were below the age of 18, of which 35,000 were unaccompanied [2,3].

Previous studies have observed differences in health and mortality between immigrants and individuals of the host population, but studies on young adult immigrants are sparse [1,4–7]. Some refugee and non-refugee immigrants coming to Sweden have been reported to have a lower all-cause mortality [8]; for example, these groups have been shown to have a lower burden of mortality due to injuries in the country of destination compared to native Danes [9] and Finns, although some results are imprecise and differ by country of origin [10]. In contrast, traffic accidents, trauma and violence have been mentioned as reasons for higher mortality in young migrants due to a possibly higher risk-prone behaviour of adolescent men compared to the host population [11–13]. Moreover, diseases with an increased mortality risk such as common mental disorders (depression, anxiety and stress-related disorders) are more prevalent among refugees than among individuals in the host population and their prevalence vary across country of birth [3,6,10,12,14–20]. This might be due to traumatic events during and before flight and could eventually lead to higher suicide rates among refugees [21].

A higher prevalence of somatic disorders such as infections, cancer, mental and cardiovascular disorders has been observed in refugees compared to Swedish-born individuals [22]. Indeed, Helgesson et al. reported a higher prevalence of somatic disorders in refugees compared to Swedish-born individuals [22]. Despite previous knowledge in this field, studies on young migrants discriminating between unaccompanied, accompanied refugees and non-refugee immigrants, and all-cause and cause-specific mortality due to suicide and external causes are lacking to date. Due to their higher risk of exposure to traumatic experiences and lack of parental support, we hypothesise that refugees in general and unaccompanied refugees in particular have a higher risk for mortality, particularly suicide mortality, as mentioned above. Further, little is known about the difference in mortality of accompanied and unaccompanied refugees that came to the host country as children, and as they make up a substantial part of international refugees, it is important to disentangle possible differences in these two populations.

There are several sociodemographic and socioeconomic factors that affect the association of migration status and mortality. When studying this association, factors such as refugee status and educational level should be taken into account. Education has previously been linked to health and mortality and is often used as a marker for socio-economic status [23–25]. Educational status has been shown to be lower in refugees and other migrants compared to Swedish-born youth [14,26]. Furthermore, in young adult refugees, a graded relationship between education and common mental disorders has been shown [14]. Refugees with compulsory school education had the highest risk of common mental disorders, followed by refugees with high school education, compared to refugees with a college or university degree [14]. The role of educational level in the association with cause-specific mortality due to suicide and external causes in young adult refugee and non-refugee immigrants has, however, not been investigated to date.

Young migrants build a heterogeneous group. Not only socioeconomic but also migration-related factors, such as country of birth, age at arrival and duration of residency, might influence the risk of mortality. Duration of stay in the host country plays a significant role in achieving good language skills and understanding the healthcare system that can contribute to a better health seeking behaviour, which in turn might have an effect on the mortality risk [27,28]. However, contrasting findings have been observed, with a longer duration of stay and earlier age at migration being associated with an increased risk of somatic disorders and anxiety disorders [29], but a lower risk of post-traumatic stress disorders [14]. A systematic review and meta-analysis concluded that migrants and refugees have a mortality advantage compared to the population of Western host countries, but the same association is unclear for more marginalised groups, such as undocumented migrants [17]. Scrutiny of the importance of migration-related factors in young migrants’ mortality risk might shed light on the pathways to worse health outcomes and hereby provide insights for the design of prevention strategies.

The aim of this study was to determine differences in all-cause and selected cause-specific mortality due to suicide and external causes in young accompanied and unaccompanied refugees and non-refugee immigrants compared to Swedish born individuals. An additional aim was to determine the role of educational level, duration of residency in the host country, age at arrival and country of birth in the associations between migrant/refugee status and mortality outcomes.

## Methods

### Study population

The study population was defined as all individuals born between 1979 and 1985, residing in Sweden and being 19–25 years old on 31 December 2004 (n = 737,606, 13.8% migrants). For migrants (n = 101,569), only those entering Sweden as children (<18 years of age) and with complete information on their reason for settlement in Sweden were eligible for inclusion (excluding those individuals the study population included 685,290, 7.6% migrants). Individuals with missing information on reason for settlement are primarily EU citizens that do not register their right of residence with the Swedish Tax Agency. We made further exclusions for those whose information on year of immigration was missing (n = 65 migrants, 0.1% of migrants). Subsequently non-refugee immigrants who did not originate from the same countries as refugees were also excluded (n = 2,867, 2.8% of all migrants). Applying these exclusion criteria, the final study population comprised 682,358 individuals, of which 633,167 were Swedish-born individuals (92.8%), 27,821 were refugees (4.1%) (2,163 unaccompanied and 25,658 accompanied) and 21,370 were non-refugee immigrants (3.1%).

We used the unique (de-identified) Swedish personal identity number to link information from several population-based registers [30]. The Longitudinal Integration Database for Health Insurance and Labour Market Studies contains data from the labour market and educational and social sectors [31]. The Longitudinal Database for Integration Studies register holds migration-related information, including reasons for settlement (e.g. refugee status). The National Patient Register (NPR) includes information on inpatient care since 1987 and for specialized outpatient care since 2001. The Cause of Death Register (CDR) comprises information on all deaths of Swedish residents since 1952. Diagnoses in the NPR and the CDR are coded according to the International Classification of Diseases, version 10 (ICD-10) [32]. The multi-generation register was established in 1961 and contains 97% complete information for mothers and 95% for fathers [33].

According to the regional ethical review board in Stockholm, Sweden, the requirement that informed consent of research subjects should be obtained has been waived due to the use of register data.

### Measures

A refugee was defined as an individual receiving a residence permit in Sweden as a refugee (according to the Geneva Convention of Refugees), or an individual who had been granted a residence permit due to being “in need of protection” or on “humanitarian grounds”. Refugees were further classified as unaccompanied or accompanied: the latter included those who had obtained residency because they were related to a family member who was a refugee according to the register, or had at least one parent in the Multi-Generation Register who had received residency in Sweden the same year or one year before the young adult refugee. Young adult refugees who did not fulfil either of these two criteria were categorized as unaccompanied. All other immigrants were categorized as non-refugee immigrants. The comparison group included young people born in Sweden.

Highest attained educational level based on years under education was measured on December 31, 2004 and categorized into compulsory school (<9 years), high school (10–12 years), college or university (>12 years) or missing. Duration of formal residency in Sweden was categorized into three groups: <5 years, 5–10 years, and >10 years. Age at arrival was grouped as follows: < = 6, 7–13, 14–16 and > = 16 years. The choice of categorisation reflects the years for different levels of schooling in Sweden.

### Outcome measures

The outcomes were defined as all-cause mortality and selected cause-specific mortality. The following selected causes were studied: suicide including deaths with undetermined intent (ICD-10: X60-84, Y10-34) and external causes consisting of accidents and injuries (ICD-10: V00-Y99). In this manuscript, mortality due to suicide and external causes will be referred to as cause-specific mortality.

### Covariates

The covariates included in the analyses, based on the directed acyclic graph (supplementary material), were I) *Socio-demographic factors*: sex, age, educational level, (all variables were measured at baseline, i.e. 31^st^ December 2004); II) *Work-related factors*: unemployment (0, 1–180, >180 days); sickness absence (0, 1–90, >90 days) and disability pension, measured in 2004; and III) *health related factors*: psychiatric (ICD F00-99) and somatic (all ICD codes except psychiatric) morbidity (in- and specialised outpatient health care) diagnosed the year prior to cohort entry, i.e. 1^st^ January until 31^st^ December 2004. The categorisation of these covariates can be seen in Table 1. Missing values in any covariate were treated as separate categories.

**Table 1:** Baseline characteristics, by refugee status, in individuals aged 19–25 years old residing in Sweden in 2004.

	Swedish-born individuals	Refugees			Non-refugee immigrants
		All	Unaccompanied	Accompanied	
All	633,167	27,821	2,163	25,658	21,370
***Sociodemographic factors at baseline***					
*Sex (n (%))*					
Women	307,336 (49)	13,013 (47)	838 (39)	12,175 (47)	10,476 (49)
Men	325,831 (51)	14,808 (53)	1,325 (61)	13,483 (53)	10,894 (51)
*Mean age (Median*, *(IQR))*	22 (20–24)	22 (20–23)	22 (20–24)	21 (20–23)	21 (20–23)
*Education (n (%))*					
Compulsory school	84,856 (13)	6,929 (25)	765 (35)	6,164 (24)	6,547 (31)
High school	383,919 (61)	14,576 (52)	788 (36)	13,788 (54)	9,084 (43)
College or university	156,036 (25)	4,892 (18)	220 (10)	4,672 (18)	2,614 (12)
Missing	8,356 (1)	1,424 (5)	390 (18)	1,034 (4)	3,125 (15)
*Country of birth (n (%))*					
Horn of Africa^1^		1,367 (5)	472 (22)	895 (3)	1,968 (9)
Afghanistan		288 (1)	120 (6)	168 (1)	576 (3)
Asia ^2^		3,228 (12)	146 (7)	3,082 (12)	4,686 (22)
Iraq		2,581 (9)	509 (24)	2,072 (8)	3,344 (16)
Iran		3,893 (14)	216 (10)	3,677 (14)	1,968 (9)
Former Yugoslavia		11,494 (41)	333 (15)	11,161 (43)	1,224 (6)
Syria		1,056 (4)	88 (4)	968 (4)	348 (2)
Others		3,914 (14)	279 (13)	3,635 (14)	7,256 (34)
*Duration of formal residency in Sweden*					
Mean number of years (SD)		11.5 (3.6)	8.0 (4.5)	11.8 (3.4)	9.8 (4.7)
0–4 years		1,613 (6)	641 (30)	972 (4)	3,384 (16)
5–10 years		9,654 (35)	790 (37)	8,864 (35)	8,268 (39)
> 10 years		16,554 (60)	732 (34)	15,822 (62)	9,718 (45)
*Family situation (n (%))*					
Married/living with partner without children^3^	6,417 (1)	1,477 (5)	112 (5)	1,365 (5)	947 (4)
Married/living with partner with children^3^	28,513 (5)	2,195 (8)	210 (10)	1,985 (8)	1,735 (8)
Single/divorced/separated/widowed without children^3^	452,435 (71)	16,712 (60)	1,622 (75)	15,090 (59)	12,446 (58)
Single/divorced/separated/widowed with children^3^	8,179 (1)	551 (2)	77 (4)	474 (2)	479 (2)
Children (≤20 years old)^3^	137,623 (22)	6,886 (25)	142 (7)	6,744 (26)	5,763 (27)
*Type of living area (n (%))*					
Big city area	221,484 (35)	12,738 (46)	1,143 (53)	11,595 (45)	12,245 (57)
Intermediate (>90,000 inhabitants)	246,851 (39)	11,089 (40)	762 (35)	10,327 (40)	6,555 (31)
Small (rural municipalities)	164,832 (26)	3,994 (14)	258 (12)	3,736 (15)	2,570 (12)
***Work-related factors at baseline*** *(n (%))*					
No unemployment *(n (%))*	455,518 (71.9)	17,327 (62.3)	1,307 (60.4)	16,020 (62.4)	14,389 (67.3)
Unemployment ≤ 180 days/year	164,147 (25.9)	9,359 (33.6)	760 (35.1)	8,599 (33.5)	6,347 (29.7)
Unemployment >180days/year	13,502 (2.1)	1,135 (4.1)	96 (4.4)	1,039 (4.0)	634 (3.0)
No sickness absence	600,306 (94.8)	26,389 (94.9)	2,041 (94.4)	24,348 (94.9)	20,643 (96.6)
Sickness absence ≤90 days/year	24,014 (3.8)	1,060 (3.8)	88 (4.1)	972 (3.8)	516 (2.4)
Sickness absence >90 days/year	8,847 (1.4)	372 (1.3)	34 (1.6)	338 (1.3)	211 (1.0)
No disability pension	621,455 (98.2)	27,358 (98.3)	2,133 (98.6)	25,225 (98.3)	20,985 (98.2)
Disability pension	11,712 (1.8)	463 (1.7)	30 (1.4)	433 (1.7)	385 (1.8)

^1^ Somalia, Eritrea and Ethiopia

^2^ except Afghanistan, Iraq, Iran and Syria

^3^ living at home.

### Statistical analyses

Crude and multivariate analyses were performed using Cox regression models of time to all-cause and cause-specific mortality during follow-up. We assessed person-years at risk by totalling the years that the individuals were living in Sweden during the follow-up period. The underlying timescale were calendar days and the entry date was defined as January 1^st^, 2005, and censoring was defined as the date of first outcome, date of emigration, or the end of follow-up (December 31^st^, 2016). Risk estimates for refugees and non-refugee immigrants were compared to those for Swedish-born individuals with regard to all-cause mortality and cause-specific mortality due to suicide and external causes. Further, we split refugees into two categories consisting of unaccompanied and accompanied refugees. To assess the role of educational level, duration of residence, age of arrival and country of birth on the outcome measures, separate analyses were conducted that were stratified for each of these factors, respectively. Further, a partial likelihood ratio test was performed in order to assess a potential effect modification between refugee status and education. We examined the associations in one crude (Model 1) and one adjusted (Model 2) regression model, which included the covariates described above. Results are presented as crude and adjusted hazard ratios (HRs and aHRs) with 95% confidence intervals (CIs). The Directed Acyclic Graph to the models of direct effects can be found as S1 Fig.

We conducted a sensitivity analysis, in which individuals who were granted residence permits due to ‘in need of protection’ and on ‘humanitarian grounds’ were excluded from the refugee category (n = 1,701) (S1 Table). Second, we conducted separate analyses in which we excluded suicide cases coded as undetermined intent (S2 Table). Last, a sensitivity analysis of the main analysis was carried out, in which we only included refugees and compared unaccompanied with accompanied refugees, also adjusting for country of birth and duration of residence (S3 Table). The reason to carry out these sensitivity analyses was to see whether the excluded participants due to ‘need of protection’ and ‘humanitarian grounds’ and ´death to undetermined intent were driving the associations that could be observed in the main analyses. Further, the third sensitivity analysis was to investigate whether unaccompanied refugees differed from accompanied refugees. Statistical analyses were conducted using SAS, v.9.4. The senior author can be contacted for details about data management and/or analyses.

### Ethical approval

This study was approved by the regional ethical board in Stockholm, Sweden (Decision 20071762–31).

## Results

Table 1 presents the population characteristics stratified by refugee status. The proportion of men and women was similar across Swedish-born individuals, refugees and non-refugee immigrants. Only when distinguishing the group of refugees into unaccompanied and accompanied refugees, there was a higher proportion of men in the unaccompanied refugees’ group. Refugees and non-refugee immigrants were slightly younger than Swedish born individuals, with mean ages ranging from 21.4 years in non-refugee immigrants to 22.0 years in Swedish born individuals. Education levels differed across the groups, with a smaller proportion of refugee and non-refugee immigrants completing college or university education, compared to Swedish-born individuals. Refugees were most often from Former Yugoslavian countries, whereas non-refugee immigrants mostly came from Asia or countries grouped as “others”. Unaccompanied refugees had a shorter mean length of formal residency in Sweden than accompanied refugees did. Most young individuals lived alone, a few were either living with their partner or were single with children living at home. Migrants were more often unemployed and living in big cities compared to their counterparts in the host country.

Table 2 displays the HRs and 95% CIs of all-cause and selected cause-specific mortality in refugees and non-refugee immigrants compared to Swedish-born individuals. After adjusting for covariates, non-refugee immigrants had the lowest statistically significant risk for all-cause mortality (aHR 0.67, 95% CI 0.56–0.80), mortality due to suicide (aHR 0.40, 95% CI 0.26–0.60) and external causes mortality (aHR 0.65, 95% CI 0.52–0.82) when compared to Swedish-born individuals. Further, accompanied refugees also showed to be protected from all-cause mortality (aHR 0.73, 95% CI 0.62–0.87), mortality due to suicide (aHR 0.61, 95% CI 0.44–0.83) and mortality due to external causes (aHR 0.72, 95% CI 0.59–0.88) compared to the Swedish-born. Even though not statistically significant, a protective trend can be observed for unaccompanied refugees. This might be due to positive confounding, i.e. some variables in the adjusted model, having a protective effect on the association.

**Table 2:** Risk of all-cause and cause-specific mortality in Swedish-born individuals and refugee and non-refugee immigrants, aged 19–25 years old residing in Sweden in 2004. Hazard ratios (HRs) with 95% confidence intervals (CIs).

	No. deaths (rate per 100,000 person-years)	Model 1^a^	Model 2^b^	Model 3^c^	Model 4^d^
	**All-cause mortality**				
Swedish-born individuals	4,097 (55.4)	1 (REF)	1 (REF)	1 (REF)	1 (REF)
Non-refugee immigrants	128 (53.3)	0.97 (0.81–1.16)	**0.55 (0.46–0.66)**	**0.67 (0.56–0.80)**	**0.67 (0.56–0.80)**
Refugees	166 (51.7)	0.92 (0.79–1.08)	**0.68 (0.58–0.80)**	**0.72 (0.62–0.85)**	**0.72 (0.62–0.85)**
Unaccompanied	16 (66.1)	1.11 (0.68–1.81)	**0.55 (0.34–0.90)**	0.67 (0.41–1.09)	0.64 (0.39–1.05)
Accompanied	150 (50.6)	0.91 (0.77–1.07)	**0.70 (0.60–0.83)**	**0.73 (0.62–0.86)**	**0.73 (0.62–0.87)**
	**Suicide**				
Swedish-born individuals	1,375 (18.6)	1 (REF)	1 (REF)	1 (REF)	1 (REF)
Non-refugee immigrants	24 (10.0)	**0.53 (0.35–0.79)**	**0.36 (0.24–0.54)**	**0.39 (0.26–0.58)**	**0.40 (0.26–0.60)**
Refugees	46 (14.3)	0.75 (0.56–1.01)	**0.59 (0.44–0.79)**	**0.59 (0.44–0.79)**	**0.61 (0.45–0.82)**
Unaccompanied	<8 (20.7)	1.02 (0.42–2.45)	0.62 (0.26–1.50)	0.65 (0.27–1.57)	0.65 (0.27–1.56)
Accompanied	41 (13.8)	**0.73 (0.53–0.99)**	**0.58 (0.43–0.79)**	**0.58 (0.43–0.79)**	**0.61 (0.44–0.83)**
	**External causes**				
Swedish-born individuals	2,657 (35.9)	1 (REF)	1 (REF)	1 (REF)	1 (REF)
Non-refugee immigrants	82 (34.2)	0.94 (0.76–1.17)	**0.62 (0.49–0.77)**	**0.63 (0.51–0.79)**	**0.65 (0.52–0.82)**
Refugees	110 (34.3)	0.93 (0.77–1.13)	**0.71 (0.59–0.86)**	**0.69 (0.57–0.84)**	**0.71 (0.59–0.86)**
Unaccompanied	11 (45.5)	1.14 (0.63–2.06)	0.67 (0.37–1.21)	0.65 (0.36–1.19)	0.65 (0.36–1.18)
Accompanied	99 (33.4)	0.91 (0.75–1.11)	**0.72 (0.59–0.87)**	**0.70 (0.57–0.85)**	**0.72 (0.59–0.88)**

^a^ Adjusted for age and sex.

^b^ Adjusted for age, sex and education.

^c^ Adjusted for age, sex, education, unemployment, sickness absence and disability pension.

^d^ Adjusted for age, sex, education, unemployment, sickness absence, disability pension, and psychiatric and somatic morbidity at baseline.

To avoid identification, groups consisting of less than 8 participants are marked with <8.

Associations between educational level and all-cause mortality were investigated by comparing each level of education in the three groups (Swedish-born, non-refugee immigrants and refugees) to Swedish-born individuals who had completed a college or university degree (Table 3). As the partial likelihood ratio test between education and refugee status indicated an interaction (p = 0.01), stratified analyses were conducted. Across all three populations, the highest adjusted risk estimate of all-cause mortality was among the Swedish-born individuals who had completed only compulsory school education (aHR 3.96, 95% CI 3.53–4.42), in contrast, risks were lower in non-refugee immigrants that completed only compulsory school education (aHR 2.48, 95% CI 1.89–3.25) and refugees of the same educational level (aHR 2.36, 95% CI 1.81–3.06). In all three groups, the adjusted risks of all-cause mortality decreased with higher level of school education.

**Table 3:** Associations between educational level and all-cause mortality 2005–2016 in Swedish-born individuals and refugee and non-refugee immigrants, aged 19–25 years old residing in Sweden in 2004. Hazard ratios (HRs) with 95% confidence intervals (CIs).

	No. deaths (rate per 100,000 person-years)	Model 1^a^	Model 2^b^
***Swedish-born individuals***			
College or university	440 (24.3)	1 (REF)	1 (REF)
High school	1,919 (42.7)	**1.77 (1.59–1.96)**	**1.48 (1.33–1.65)**
Compulsory school	1,457 (146.7)	**5.96 (5.34–6.65)**	**3.95 (3.53–4.42)**
Missing	281 (299.1)	**12.49 (10.72–14.55)**	**5.13 (4.28–6.14)**
***Non-refugee immigrants***			
College or university	<8 (23.8)	1.00 (0.47–2.11)	0.96 (0.45–2.02)
High school	41 (39.9)	**1.70 (1.24–2.35)**	**1.39 (1.01–1.92)**
Compulsory school	61 (82.2)	**3.45 (2.63–4.52)**	**2.48 (1.89–3.25)**
Missing	19 (56.5)	**2.70 (1.70–4.27)**	**2.13 (1.34–3.39)**
***Refugees***			
College or university	10 (17.8)	0.76 (0.41–1.43)	0.73 (0.39–1.37)
High school	74 (43.9)	**1.81 (1.42–2.32)**	**1.42 (1.11–1.82)**
Compulsory school	66 (82.5)	**3.35 (2.58–4.34)**	**2.36 (1.81–3.06)**
Missing	16 (98.8)	**4.44 (2.69–7.32)**	**2.74 (1.66–4.54)**

^a^ Adjusted for age and sex.

^b^ Adjusted for age, sex, unemployment, sickness absence, disability pension, and psychiatric and somatic morbidity at baseline.

To avoid identification, groups consisting of less than 8 participants are marked with <8.

The educational levels correspond to years under education. Hence, <9 years of education for compulsory school, 10–12 years for high school and >12 years for university.

Table 4 provides the results of examining the effect of duration of formal residency and age at first immigration on all-cause mortality. Among non-refugee immigrants, those with less than 5 years of formal residency in Sweden had the lowest risk of all-cause mortality (aHR 0.37, 95% CI 0.21–0.64) when compared to Swedish-born individuals. In refugees, the risk was lowest in those with 5 and 5–10 years of formal residency in Sweden (aHR 0.57, 95% CI 0.32–1.01; aHR 0.56, 95% CI 0.42–0.74, respectively). Refugee and non-refugee immigrants that stayed in Sweden longer than 10 years did not show a reduction in all-cause mortality risk, although a protective trend can be observed.

**Table 4:** Risk of all-cause mortality by refugee status, duration of formal residency in Sweden, and age at first immigration respectively. Individuals aged 19–25 years old residing in Sweden in 2004. Hazard ratios (HRs) with 95% confidence intervals (Cis).

	No. deaths (rate per 100,000 person-years)	Model 1^a^	Model 2^b^
**Duration of formal residency**			
Swedish-born individuals	4,097 (55.4)	1 (REF)	1 (REF)
***Non-refugee immigrants***			
<5 years	13 (34.3)	0.65 (0.38–1.12)	**0.37 (0.21–0.64)**
5–10 years	41 (44.4)	0.81 (0.59–1.10)	**0.53 (0.39–0.73)**
>10 years	74 (67.4)	1.21 (0.96–1.53)	0.93 (0.74–1.17)
***Refugees***			
<5 years	12 (64.6)	1.11 (0.63–1.96)	0.57 (0.32–1.01)
5–10 years	47 (41.9)	**0.75 (0.56–1.00)**	**0.56 (0.42–0.74)**
>10 years	107 (56.3)	1.01 (0.83–1.22)	0.86 (0.71–1.04)
**Age at first immigration**			
Swedish-born individuals	4,097 (55.4)	1 (REF)	1 (REF)
***Non-refugee immigrants***			
0–6 years	25 (64.2)	1.18 (0.80–1.76)	0.92 (0.62–1.36)
7–13 years	61 (59.8)	1.07 (0.83–1.38)	0.80 (0.62–1.04)
14–16 years	24 (45.6)	0.82 (0.55–1.22)	**0.51 (0.34–0.76)**
>16 years	18 (38.8)	0.74 (0.47–1.18)	**0.44 (0.27–0.70)**
***Refugees***			
0–6 years	33 (57.3)	1.04 (0.74–1.46)	0.91 (0.65–1.28)
7–13 years	100 (50.9)	0.91 (0.75–1.11)	**0.75 (0.61–0.91)**
14–16 years	20 (43.7)	0.77 (0.50–1.19)	**0.54 (0.35–0.84)**
>16 years	13 (61.6)	1.05 (0.61–1.80)	**0.57 (0.33–0.99)**

^a^ Adjusted for age and sex.

^b^ Adjusted for age, sex, education, unemployment, sickness absence, disability pension, and psychiatric and somatic morbidity at baseline.

When non-refugee immigrants and refugees were categorised according to age at the time of immigration, non-refugee immigrants aged 14–16 years and > 16 years when entering Sweden (aHR 0.53, 95% CI 0.36–0.80; aHR 0.44, 95% CI 0.27–0.70) and refugees aged 7–13 years (aHR 0.78, 95% CI 0.64–0.95),14–16 years (aHR 0.58, 95% CI 0.37–0.90) and > 16 years (aHR 0.57, 95% CI 0.33–0.99) were at significantly lower risk of all-cause mortality than Swedish-born individuals.

Table 5 shows hazard ratios for all-cause mortality by immigrant status (combining refugee and non-refugee immigrants) and country of birth. The lowest risk of all-cause mortality was among those immigrating from Iraq (aHR 0.37, 95% CI 0.25–0.55), Asia (aHR 0.55, 95% CI 0.40–0.76) and Former Yugoslavia (aHR 0.62, 95% CI 0.48–0.79) compared to Swedish-born. Most of the remaining countries of birth, with the exception of Iran, indicated a lower risk of all-cause mortality for immigrants, but there were no statistically significant differences.

**Table 5:** Risk of all-cause mortality by refugee status and country of birth. Individuals aged 19–25 years old residing in Sweden in 2004. Hazard ratios (HRs) with 95% confidence intervals (CIs).

	No. deaths (rate per 100,000 person-years)	Model 1^a^	Model 2^b^
Swedish-born individuals	4,097 (55.4)	1 (REF)	1 (REF)
***Non-refugee or refugee immigrants***			
Horn of Africa^c^	30 (85.1)	**1.49 (1.04–2.13)**	0.94 (0.66–1.35)
Afghanistan	< 8 (60.8)	1.02 (0.46–2.26)	0.65 (0.29–1.46)
Asia^d^	38 (41.8)	0.77 (0.56–1.06)	**0.55 (0.40–0.76)**
Iraq	24 (35.1)	**0.62 (0.41–0.92)**	**0.37 (0.25–0.55)**
Iran	47 (71.1)	1.26 (0.95–1.68)	1.12 (0.84–1.49)
Former Yugoslavia	63 (42.3)	**0.77 (0.60–0.98)**	**0.62 (0.48–0.79)**
Syria	< 8 (30.3)	0.55 (0.23–1.32)	0.42 (0.17–1.00)
Others	81 (64.9)	1.17 (0.94–1.46)	0.90 (0.72–1.13)

^a^ Adjusted for age and sex.

^b^ Adjusted for age, sex, education, unemployment, sickness absence, disability pension, and psychiatric and somatic morbidity at baseline.

^c^ Somalia, Eritrea and Ethiopia.

^d^ Except Afghanistan, Iraq, Iran and Syria.

To avoid identification, groups consisting of less than 8 participants are marked with <8.

### Sensitivity analysis

In a sensitivity analysis, refugees who were granted residence permit due to “in need of protection” and on “humanitarian grounds” were excluded. The risk of all-cause mortality in non-refugee immigrants showed similar results in both the main analysis in Table 2 and the sensitivity analysis. Further, the estimates for suicide and mortality due to undetermined intent were comparable. Finally, when only comparing unaccompanied refugees to accompanied refugees in a sensitivity analysis, no effect of adjustment for country of birth or duration of residency could be found.

## Discussion

### Main findings

The adjusted rates of all-cause mortality and mortality due to suicide and due to external causes were lower in young migrants with no differences between refugees (unaccompanied and accompanied) and non-refugee immigrants. There was a clear gradient with educational level, duration of residency and age at arrival for both migrant groups: estimates increased with decreasing education and age at immigration and increasing length of residency. The mortality risk estimates of migrants varied by country of birth.

### All-cause and cause-specific mortality in migrants and refugees

Our finding that the risk of all-cause mortality was lower in young non-refugee immigrants compared to Swedish-born individuals is in line with previous studies and a meta-analysis and systematic review including all age groups [5,17,20]. This comparability might be due to the similarity in age and country of origin of the study population and outcome definition used for mortality and suicide [5,20]. However, this is contradicting the previously reported U-shaped relationship between mortality and age, reporting higher mortality rates in young migrants [34]. The difference might be explained by the country of residence, as UK, US and France as host countries are examined and the inclusion of a larger age group that ranges from 5 years to 85 years and older. Hence, we were unable to support the U-shape relationship between age at immigration and mortality. Further, reasons for a lower mortality in young age groups in our study compared to the mentioned study could be the difference of countries of origin. While our study includes countries and regions refugees stem from such as the Horn of Africa, Afghanistan, Asia, Iraq, Iran, Former Yugoslavia and Syria, the study reporting the U-shape included countries such as Algeria, Italy, Spain, Tunisia, Turkey and the UK among others [34]. These findings are consistent with the hypothesis of a ‘healthy migrant effect’ [3,5,7], which claims that migrants experience better health outcomes relative to both the majority population in the host country and individuals who remain in their country of origin. The ‘healthy migrant effect’ here describes therefore a healthy selection effect arguing that migrants with high morbidity levels would not be able to overcome the challenges of migration. The ‘healthy migrant effect’ should be related to the ‘health transition theory’, which during the first period of migration relates to the transition or shift from communicable to non-communicable diseases [35]. In the second period, the theory relates to changes in health outcomes related to social, cultural and behavioural determinants of health [35]. Following this theory, it can be expected to see a lower mortality in young adults coming from a country with a higher incidence of infectious diseases to Sweden, as non-communicable diseases often are of degenerative nature with an increased prevalence in older ages [35].

Other explanations for the lower mortality risk include favourable health behaviour and outmigration selection, as well as cultural buffering which suggests that migrants have a stronger bond to family and extended kinship which might act as a health-promoting factor [5,34]. Moreover, other reasons might contribute to the lower mortality rates in immigrants resettling in high income countries such as the fact that infectious and maternal health issues can be treated quickly and effectively in the host countries and there is a lag between adopting a lifestyle that gives rise to risk factors for ischaemic heart disease for instance [36].

The findings regarding mortality due to suicide are consistent with a recent study reporting a lower suicide risk in refugees with residence permit compared to native Swedes [20]. This is, however, contradicting a recent report on an 8-fold higher suicide risk in unaccompanied minors seeking asylum compared to the general population of the same age in Sweden [37].

Our results regarding cause-specific mortality due to external causes are in line with a study conducted in Finland [10], which observed a higher mortality in native adult Finns due to their excessive alcohol consumption and the following possible deaths due to external causes, such as accidents compared to migrants coming to Finland [10]. However, a study conducted in Sweden found a higher mortality due to external causes, which include but are not limited to higher rates of assault and uncertain housing, particularly in undocumented migrants [13].

### Education and all-cause mortality

We found that lower education was a risk factor for all-cause mortality in both refugees and non-refugee immigrants, as well as in Swedish-born individuals. These findings are consistent with previous studies which have shown that lower socio-economic status (e.g. measured as educational level) is associated with subsequent mortality [23–25]. Reasons for a higher mortality in lower educated individuals could be explained by a different health seeking behaviour, lower health literacy and less access to social support and monetary resources such as job stability, as well as less freedom towards lifestyle choices [25]. The most obvious mortality disadvantage was, however, not found in any migrant group but in young Swedish-born individuals with low education, deserving further scrutiny.

### Duration of residency and age at arrival in the host country and subsequent all-cause mortality

Our findings suggest that the longer the formal residency in Sweden and the younger the age at arrival, the higher the all-cause mortality in young non-refuge immigrants and refugees. We could replicate findings of a study that focussed on migrants in Norway [38]. It is hypothesised that the positive health selection of immigrants deteriorates after prolonged stay in the host country and thus, mortality rates increase to the same level as the native population [38]. Besides the deterioration of the health selection, a changed lifestyle and the acculturation to the host culture might be reasons for the change in all-cause mortality [27,28].

### Country of birth and all-cause mortality

Young adult refugee and non-refugee immigrants from Iraq, Asia and Former Yugoslavia had the lowest risk of all-cause mortality, whereas immigrants from Iran, Afghanistan, Syria and the horn of Africa had a similar mortality risk as their Swedish-born peers. Our findings can be related to a study conducted in Denmark, in which all-cause mortality was higher in adult women from Sub-Saharan Africa compared to Danish-born individuals [9]. Even though the regions of origin are different with Sub-Saharan Africa and the Horn of Africa and there are slightly different mean ages in the population, the comparison might still be valuable. Reasons behind the loss of a mortality advantage among migrants from some countries might be both related to health behaviour, knowledge about and use of the health care and social insurance system as well as differential social marginalisation experiences [17].

### Strengths and limitations

The strengths of this study include the use of a large dataset based on register data, leading to practically no loss to follow-up, high validity of data [31,39] and the ability to adjust for a wide range of covariates. This allowed the analyses of subgroups in relation to a rare outcome measures over a long follow-up period, which adds an important contribution to the sparse existing scientific knowledge base on young migrants.

Some limitations also have to be mentioned. Despite the large size of the study population, statistical power was decreased in some analyses, particularly within the group of unaccompanied refugees. Unfortunately, the statistical power was reduced when using specific causes of deaths, given the young age of the study participants. Hence, the focus should be primarily on all-cause mortality, mortality due to suicide and external causes. Further, information on primary health care diagnoses for the detection of somatic and psychiatric morbidities could not be included in the measurement of covariates, which was based on diagnoses in specialised health care. Hence, primarily cases of a high medical severity were covered, which might have led to an underestimation of the effect of morbidity at baseline. This underestimation might be stronger for immigrants and refugees from some countries, as some immigrant groups are known to underutilise specialised health care [40,41]. Hence, this underestimation might lead to the fact that the observed association is stronger than the real association. However, another study found that immigrants from Somalia Former Yugoslavia and Turkey over utilise emergency care in Denmark [42]. Additionally, there might be more underreporting of suicidal behaviour in refugee and non-refugee immigrants due to stronger stigmatisation in countries immigrants stem from compared to Westernised host countries [43]. We diminished a potential bias due to stronger underreporting in immigrants by including external causes as well and not only death due to suicide. Despite a considerable proportion of migrants excluded from the study population, these exclusions did not result in selection bias due to the fact that they were due to age restrictions and restrictions regarding country of origin. The latter responds to the aim of the study to compare refugees with non-refugee migrants from similar countries of birth. Moreover, in addition to the commonly used definition of refugees, we included refugees that were granted permission due to “the need of protection” or “humanitarian grounds”. Sensitivity analyses did not reveal any differences in mortality risk in these groups compared to the main analyses.

## Conclusion

This is one of the first studies to examine the risk of all-cause and cause-specific mortality due to suicide and external causes in young adult refugees and non-refugee immigrants, whilst distinguishing the group of refugees into unaccompanied and accompanied. While the risk of all-cause and cause-specific mortality was generally lower in the migrant groups compared to Swedish-born individuals, this mortality seems to be higher in refugees and non-refugee immigrants with low education. These risk groups need to be addressed in intervention studies attempting to lower the risk of all-cause and cause-specific mortality in young adult refugee and non-refugee immigrants.

## Supporting information

S1 Fig Hypothesised directed acyclic graph on the association between migration status and mortality. Please note that the hypothesised DAG represents simplified associations for confounders and mediators, as not all pathways between the covariates are displayed.
(TIF)

S1 Table Risk of all-cause mortality in Swedish-born individuals by refugee status, in individuals aged 19–25 years old residing in Sweden in 2004. Hazard ratios (HRs) with 95% confidence intervals (CIs). Excluded from the refugee category are individuals who were granted residence permits due to ‘in need of protection’ and on ‘humanitarian grounds’. ^a^ Adjusted for age and sex. ^b^ Adjusted for age, sex, education, unemployment, sickness absence, disability pension at baseline, and psychiatric and somatic morbidity in 2004.
(DOCX)

S2 Table Risk of suicide in Swedish-born individuals and refugee and non-refugee immigrants, aged 19–25 years old residing in Sweden in 2004. Hazard ratios (HRs) with 95% confidence intervals (CIs). Excluded from the suicide outcome are undetermined cases (i.e. ICD-codes Y10-34). ^a^ Adjusted for age and sex. ^b^ Adjusted for age, sex, education, unemployment, sickness absence, disability pension at baseline, and psychiatric and somatic morbidity in 2004.
(DOCX)

S3 Table Risk of all-cause mortality in refugees, aged 19–25 years old residing in Sweden in 2004. **Hazard ratios (HRs) with 95% confidence intervals (CIs)**. ^a^ Adjusted for age and sex, ^b^ Adjusted for age, sex, education, unemployment, sickness absence, disability pension at baseline, and psychiatric and somatic morbidity in 2004. ^c^ Model 2 with additional adjustments for country of birth and length of residency.
(DOCX)
