# Supplementary figures and images for: Differences in all-cause and cause-specific mortality due to external causes and suicide between young adult refugees, non-refugee immigrants and Swedish-born young adults: The role of education and migration-related factors
Source: PLoS One. 2022 Dec 20;17(12):e0279096. doi: 10.1371/journal.pone.0279096 (PMC9767339; doi:10.1371/journal.pone.0279096)

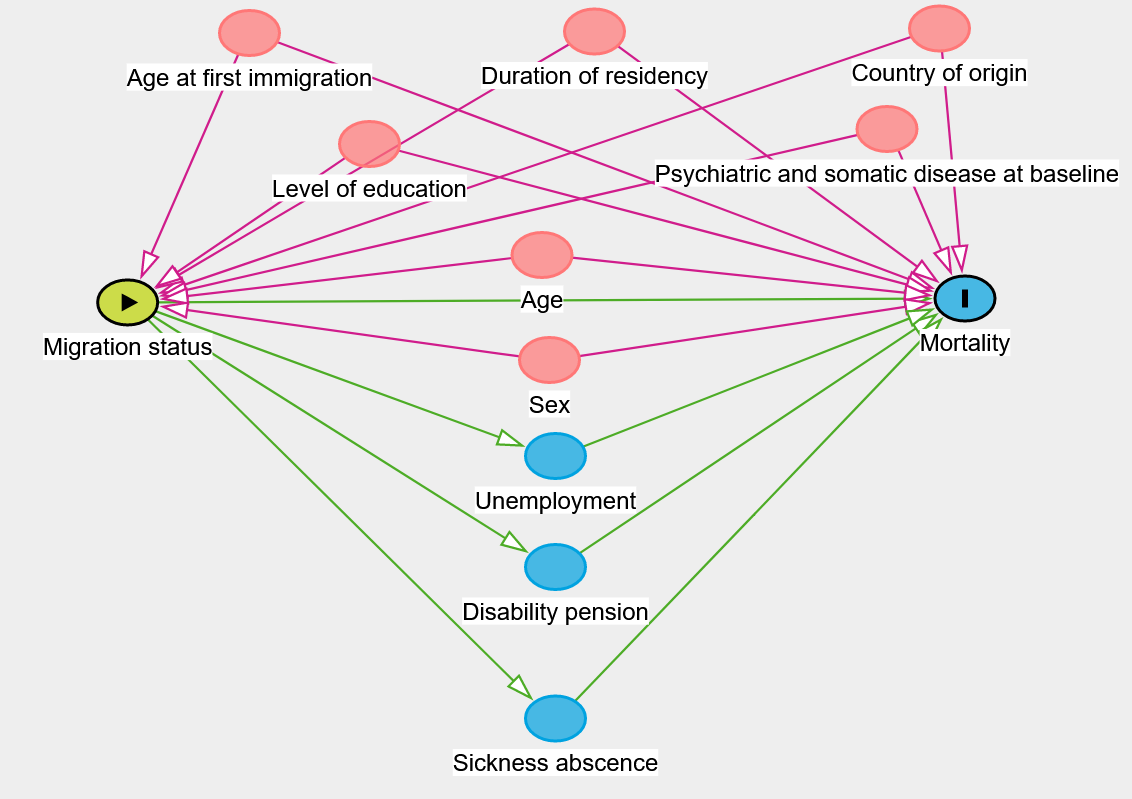

Supplement: S1 Fig — Please note that the hypothesised DAG represents simplified associations for confounders and mediators, as not all pathways between the covariates are displayed. (TIF) [file pone.0279096.s001.tif]
