# Supplementary material for: Differences in all-cause and cause-specific mortality due to external causes and suicide between young adult refugees, non-refugee immigrants and Swedish-born young adults: The role of education and migration-related factors
Source: PLoS One. 2022 Dec 20;17(12):e0279096. doi: 10.1371/journal.pone.0279096 (PMC9767339; doi:10.1371/journal.pone.0279096)
Supplement: S1 Table — Hazard ratios (HRs) with 95% confidence intervals (CIs). Excluded from the refugee category are individuals who were granted residence permits due to ‘in need of protection’ and on ‘humanitarian grounds’. a Adjusted for age and sex. b Adjusted for age, sex, education, unemployment, sickness absence, disability pension at baseline, and psychiatric and somatic morbidity in 2004. (DOCX) [file pone.0279096.s002.docx]

Table S1 Risk of all-cause mortality in Swedish-born individuals by refugee status, in individuals aged 19-25 years old residing in Sweden in 2004. Hazard ratios (HRs) with 95% confidence intervals (CIs). Excluded from the refugee category are individuals who were granted residence permits due to ‘in need of protection’ and on ‘humanitarian grounds’.

|  | **N (rate per 100,000 person-years)** | **Model 1^a^** | **Model 2^b^** |
| --- | --- | --- | --- |
| Swedish-born individuals | 4,097 (55.4) | 1 (REF) | 1 (REF) |
| Non-refugee immigrants | 128 (53.3) | 0.97 (0.81 - 1.15) | **0.69 (0.58 - 0.83)** |
| Refugees | 9 (46.2) | 0.84 (0.43 - 1.61) | 0.71 (0.37 - 1.38) |
| Unaccompanied | 0 (0.0) | N/A | N/A |
| Accompanied | 9 (48.4) | 0.88 (0.45 - 1.68) | 0.76 (0.39 - 1.46) |

^a^ Adjusted for age and sex
^b^ Adjusted for age, sex, education, unemployment, sickness absence, disability pension at baseline, and psychiatric and somatic morbidity in 2004
