# Supplementary material for: Differences in all-cause and cause-specific mortality due to external causes and suicide between young adult refugees, non-refugee immigrants and Swedish-born young adults: The role of education and migration-related factors
Source: PLoS One. 2022 Dec 20;17(12):e0279096. doi: 10.1371/journal.pone.0279096 (PMC9767339; doi:10.1371/journal.pone.0279096)
Supplement: S2 Table — Hazard ratios (HRs) with 95% confidence intervals (CIs). Excluded from the suicide outcome are undetermined cases (i.e. ICD-codes Y10-34). a Adjusted for age and sex. b Adjusted for age, sex, education, unemployment, sickness absence, disability pension at baseline, and psychiatric and somatic morbidity in 2004. (DOCX) [file pone.0279096.s003.docx]

Table S2 Risk of suicide in Swedish-born individuals and refugee and non-refugee immigrants, aged 19-25 years old residing in Sweden in 2004. Hazard ratios (HRs) with 95% confidence intervals (CIs). Excluded from the suicide outcome are undetermined cases (i.e. ICD-codes Y10-34).

|  | **N (rate per 100,000 person-years)** | **Model 1^a^** | **Model 2^b^** |
| --- | --- | --- | --- |
|  | **Suicide (X60-84 only)** | | |
| Swedish-born individuals | 1,030 (13.9) | 1 (REF) | 1 (REF) |
| Non-refugee immigrants | 16 (6.7) | **0.47 (0.29 - 0.77)** | **0.37 (0.23 - 0.61)** |
| Refugees | 34 (10.6) | 0.74 (0.53 - 1.05) | **0.63 (0.45 - 0.89)** |
| Unaccompanied | 5 (20.7) | 1.37 (0.57 - 3.29) | 0.94 (0.39 - 2.28) |
| Accompanied | 29 (9.8) | **0.69 (0.48 - 1.00)** | **0.60 (0.41 - 0.86)** |
|  | **Undetermined (Y10-34 only)** | | |
| Swedish-born individuals | 345 (4.7) | 1 (REF) | 1 (REF) |
| Non-refugee immigrants | 8 (3.3) | 0.70 (0.35 - 1.42) | **0.45 (0.22 - 0.91)** |
| Refugees | 12 (3.7) | 0.78 (0.44 - 1.38) | **0.55 (0.31 - 0.98)** |
| Unaccompanied | 0 (0.0) | N/A | N/A |
| Accompanied | 12 (4.0) | 0.85 (0.48 - 1.50) | 0.62 (0.35 - 1.11) |

^a^ Adjusted for age and sex
^b^ Adjusted for age, sex, education, unemployment, sickness absence, disability pension at baseline, and psychiatric and somatic morbidity in 2004
