# Supplementary material for: Differences in all-cause and cause-specific mortality due to external causes and suicide between young adult refugees, non-refugee immigrants and Swedish-born young adults: The role of education and migration-related factors
Source: PLoS One. 2022 Dec 20;17(12):e0279096. doi: 10.1371/journal.pone.0279096 (PMC9767339; doi:10.1371/journal.pone.0279096)
Supplement: S3 Table — Hazard ratios (HRs) with 95% confidence intervals (CIs). a Adjusted for age and sex, b Adjusted for age, sex, education, unemployment, sickness absence, disability pension at baseline, and psychiatric and somatic morbidity in 2004. c Model 2 with additional adjustments for country of birth and length of residency. (DOCX) [file pone.0279096.s004.docx]

Table S3 Risk of all-cause mortality in refugees, aged 19-25 years old residing in Sweden in 2004. Hazard ratios (HRs) with 95% confidence intervals (CIs).

| **Refugee status** | **N (rate per 100,000 person-years)** | **Model 1^a^** | **Model 2^b^** | **Model 3^c^** |
| --- | --- | --- | --- | --- |
| Unaccompanied | 16 (66.1) | 1 (REF) | 1 (REF) | 1 (REF) |
| Accompanied | 150 (50.6) | 0.82 (0.49 - 1.38) | 0.99 (0.59 - 1.68) | 1.00 (0.56 - 1.80) |

^a^ Adjusted for age and sex,
^b^ Adjusted for age, sex, education, unemployment, sickness absence, disability pension at baseline, and psychiatric and somatic morbidity in 2004
^c^ Model 2 with additional adjustments for country of birth and length of residency
